# Testing the Feasibility of Virtual Reality With Older Adults With Cognitive Impairments and Their Family Members Who Live at a Distance

**DOI:** 10.1093/geroni/igab014

**Published:** 2021-07-01

**Authors:** Tamara Afifi, Nancy L Collins, Kyle Rand, Ken Fujiwara, Allison Mazur, Chris Otmar, Norah E Dunbar, Kathryn Harrison, Rebecca Logsdon

**Affiliations:** 1 Department of Communication, University of California Santa Barbara, USA; 2 Department of Psychological and Brain Sciences, University of California Santa Barbara, USA; 3 Rendever, Boston, Massachusetts, USA; 4 Department of Psychology, National Chung Cheng University, Taiwan; 5 Corporate Applications, Blizzard Entertainment, Irvine, California, USA; 6 Department of Psychosocial and Community Health, University of Washington, Seattle, USA

**Keywords:** Dementia, Family relationships, Livestreaming, Networking, Virtual reality

## Abstract

**Background and Objectives:**

This study tests the feasibility of using virtual reality (VR) with older adults with mild cognitive impairment (MCI) or mild-to-moderate dementia with a family member who lives at a distance.

**Research Design and Methods:**

21 residents in a senior living community and a family member (who participated in the VR with the older adult from a distance) engaged in a baseline telephone call, followed by 3 weekly VR sessions.

**Results:**

Residents and family members alike found the VR safe, extremely enjoyable, and easy to use. The VR was also acceptable and highly satisfying for residents with MCI and dementia. Human and automated coding revealed that residents were more conversationally and behaviorally engaged with their family member in the VR sessions compared to the baseline telephone call and in the VR sessions that used reminiscence therapy. The results also illustrate the importance of using multiple methods to assess engagement. Residents with dementia reported greater immersion in the VR than residents with MCI. However, the automated coding indicated that residents with MCI were more kinesically engaged while using the VR than residents with dementia.

**Discussion and Implications:**

Combining networking and livestreaming features in a single VR platform can allow older adults in senior living communities to still travel, relive their past, and engage fully with life *with* their family members, despite geographical separation and physical and cognitive challenges.

**Translational Significance:** Combining networking and livestreaming features in a single virtual reality platform can allow older adults in senior living communities to still travel, relive their past, and engage fully with life with their family members, despite geographical separation and physical and cognitive challenges.

Because people are living longer, the number of people with Alzheimer’s disease (AD) and Alzheimer’s disease-related dementias (ADRD) is expanding exponentially, affecting approximately 5.4 million Americans (Centers for Disease Control and Prevention, 2020). Given that most older adults with dementia eventually end up in long-term care, there is an urgent demand for senior living communities to provide the type of quality care that older adults with dementia and their family members desperately want and deserve (Sury et al., 2013). Unfortunately, even if senior living communities provide high-quality care, many residents find themselves feeling lonely, isolated, and disconnected from their family and the rest of the world which, in turn, can dramatically diminish mental health and increase emergency hospitalizations and further cognitive deficits (Burgener et al., 2015). In addition, adult children increasingly do not live near their parents and are managing dual-career families of their own, making it incredibly difficult for them to care for their parents (Cagle & Munn, 2012). Adult children often experience anxiety and feel as if their caregiving is inadequate, especially if their parent has dementia (Koenig et al., 2014; Sury et al., 2013). Coronavirus disease 2019 (COVID-19) has undoubtedly exacerbated all of these feelings of anxiety, distress, and isolation on the part of the older adults and their family.

New technologies are necessary that can help reduce the emotional burden of AD/ADRD for older adults and their family members. Virtual reality (VR) technology can provide an innovative, drug-free, and affordable solution to these challenges by enabling older adults in senior living communities to maintain important family relationships, engage fully with life, and reconnect with their past (see also Moyle et al., 2018; Sayma et al., 2020). There is neurological, behavioral, and physiological evidence that virtual environments allow people to feel the emotional presence of others in ways that surpass their location in space (Biocca et al., 2003). Unfortunately, little research has examined the impact of VR on older adults’ social relationships, potentially because of the lack of advanced communication features of most VR technology for older adults.

VR can allow multiple people to engage in VR activities together simultaneously, even if they live in different geographical locations. Preliminary testing of the VR program, Rendever, among groups of residents without cognitive impairments showed that the technology improved mood, energy, emotional and social well-being, and physical health (Lin et al., 2018). However, its remote capabilities with older adults (regardless of their level of cognitive impairment) remain to be tested. Rendever is unique from other VR platforms for older adults because it uses networking technology that allows everyone on the network to experience the same content simultaneously— enabling group travel, co-viewing of virtual photos and videos, and other experiences. This VR platform also includes a live communication component that enables people to speak with each other remotely in the virtual world even if they are geographically separated, allowing for the maintenance of crucial social bonds. In the current study, the feasibility of using this VR platform with residents with mild cognitive impairment (MCI) or mild-to-moderate dementia and a family member who lives at a distance is evaluated.

## The Feasibility of VR With Older Adults With Cognitive Impairments

VR has the potential to alter the way older adults feel about their relationships and the world around them. VR “is an experiential interface in which the components of perception (visual, tactile, and kinesthetic) are the bases for interactivity, encouraging a sense of ‘being there’—that is, the sensation of being actually inside the virtual environment” (Optale et al., 2010, p. 349). Residents in senior living communities are often separated from the rest of society socially and spatially (Lin et al., 2018). VR is different than other forms of technology because of telepresence or immersion into a virtual world. Rather than simply chatting with family via a video screen, VR provides rich sensory stimuli that can make older adults feel as if they are actually inside the virtual world engaging in real activities with their loved ones (Cummings & Bailenson, 2016). VR could allow older adults with cognitive and/or physical impairments to continue to grow, experience new sensations, travel, and live life outside senior living communities (see also Appel, Appel, et al., 2020; Appel, Kisonas, et al., 2020).

The immersive nature of VR promotes greater engagement with the stimulus. Engagement is a multifaceted concept describing characteristics of an experience that draw people into a task. When people experience elevated engagement, they are intensely focused and curious about novel stimuli, they feel challenged and lose awareness of time, and experience intrinsic motivation (Jensen et al., 2016). Engagement is related to behaviors that are often called “immediacy” or “involvement” by nonverbal communication experts because it is part of the “approach” behaviors that demonstrate liking and positive regard (Guerrero, 2005). Engagement is often measured with self-reports that capture a retrospective summary of an experience, but Jensen and colleagues (2016) argue that coding behaviors as a measure of engagement allows for the measurement of engagement *during* the experience while it is actually happening rather than after the fact. In addition, many of the behaviors associated with engagement (e.g., immediacy in the voice, arousal) are often difficult for individuals to recall and may occur outside the individuals’ awareness (see Derrick et al., 2011). Finally, self-report measures are subjective and may be susceptible to self-report biases such as acquiescence and desirability, whereas coded behaviors, on the other hand, are relatively more objective (Jensen et al., 2016). Consequently, we employ a combination of self-reports of immersion and human and automated coding of nonverbal markers of kinesic (e.g., wide gestures, trunk movement, reaching for objects), vocalic (e.g., animated voice), and facial engagement (e.g., smiling, frowning), as well as conversational engagement with the family member while using the VR.

Virtual worlds could also create a feeling of security for residents with dementia, who are anxious without their family, and for their adult children, who long for a connection with the parent they used to know. *Social presence* is the feeling of being with another person in a virtual environment (Biocca et al., 2003). It allows people to transcend their location in space and feel as if they are with each other psychologically (Lombard & Ditton, 1999, unpublished manuscript). VR activates psychological processes in the brain that become stimulated when people are focused on each other (Biocca et al., 2003; Cummings & Bailenson, 2016). A component of social presence is the notion of *copresence* or “the degree to which the observer believes he/she is not alone and secluded, their level of peripheral or focal awareness of the other, and their sense that the other is peripherally or focally aware of them” (Harms & Biocca, 2004, p. 1). VR could help residents and their adult children maintain essential emotional bonds, especially if their children do not live near the senior living community. Adult children who care for their parent from a distance worry about their parent’s care (Sury et al., 2013) and have been shown to have similar levels of distress to siblings who are local, but report greater dissatisfaction with information they receive about their parent’s care (Thompsell & Lovestone, 2002). Engaging in VR with one’s parent could combat these adverse effects of geographic distance.

VR has been used successfully with older adults with MCI and mild-to-moderate AD/ADRD to enhance cognition, navigation, spatial orientation, attention, spatial memory, mobility, and balance (Manera et al., 2016; McEwen et al., 2014; Repetto et al., 2016). Research on residents with and without dementia suggests that VR is safe, enjoyable, and can induce positive affect (Appel, Appel, et al., 2020; Appel, Kisonas, et al., 2020). For instance, virtual environments have been found to increase joy and relaxation in residents and reduce anxiety and sadness (Angelini et al., 2015). Researchers have also tested sustained attention while using VR in a target finding task (i.e., correctly identifying certain human images in pictures), compared to a paper version of the same task, among residents with MCI and dementia (Manera et al., 2016). Residents, regardless of the level of cognitive impairment, found the VR enjoyable and reported high feelings of security and low levels of discomfort and anxiety using it. Approximately 70% of the participants also preferred the VR condition over the paper condition. In addition, residents with apathy were more interested in the VR condition than the paper condition, compared to residents who were non-apathetic.

Little research, however, has examined the impact of VR on older adults’ social relationships, regardless of their level of cognitive impairment. No research, to our knowledge, has examined shared VR experiences with older adults, with or without cognitive impairments, and their family members. What is often missing from the VR designed for older adults is sophisticated communication and networking functions that would make this possible. The ability to reach out and “touch” the family photos and travel back in time to one’s childhood home and other familiar locations with one’s adult child while sharing stories about these experiences should facilitate positive engagement with the technology and conversational engagement with the other person—for both the parent and the adult child. VR has been used with older adults without cognitive impairments for reminiscence therapy and shown that the number of memories generated is greater with familiar than unfamiliar settings (Chapoulie et al., 2014). Other researchers have found that VR can enhance autobiographical memory in older adults with MCI (Benoit, 2015) and that VR can improve memory in older adults with MCI more than music therapy (Optale et al., 2010). The current study extends this work by exploring whether VR focused on past memories can be used with residents with MCI and dementia *and their family members*, who can reminisce about these experiences together remotely.

Residents with dementia long to maintain family connections, have control over their lives, and feel as if their lives still have meaning (Eriksen et al., 2016; Moyle et al., 2011). VR could provide an important means through which they maintain their relationships with family and reclaim their past. Adult children typically provide a strong sense of familiarity and security for residents with dementia (Moyle et al., 2011). Using VR from a distance with an older adult parent in a senior living community could also benefit adult children by reducing their guilt about their parent’s quality of life. However, we first need to show that VR can be used with residents with dementia and that the software and remote capabilities work. Thus, this project involves a feasibility study of VR with residents with MCI or mild-to-moderate dementia and their adult children (or other close family members) who live at a distance to evaluate (a) the acceptability, engagement, and usability challenges of the long-distance and networking features of VR with this population, and (b) whether residents with MCI and AD/ADRD differ in their acceptability, engagement, and ability to use it.

## Method

### Participants

Data were collected from 21 family dyads from one senior living community (Maravilla, Santa Barbara, CA) in August 2019 to March 2020. *Residents* ranged in age from 54 to 94 years old (*M* = 83.10, *SD* = 9.76). The majority were female (18 females, three males) and identified as White (*n* = 19). The residents were well educated, with most having at least a Bachelor’s degree (72%). Mini-Mental State Examination-2 (MMSE-2) scores ranged from 13 to 26 (*M* = 22.19, *SD* = 3.72). MCI was categorized as scores 25–26, mild dementia as 21–24, and moderate dementia as 13–20 (see also Vertesi et al., 2001). The MMSE is one of the most commonly used assessments of cognitive impairment and is often used to screen older adults for clinical trials. The MMSE-2 provides revised questions and is more sensitive to detection of MCI. Nine residents had MCI, four had mild dementia, and eight had moderate dementia (see Supplementary Table 1). Three of the residents with moderate dementia had a spouse living with them. Two of these residents wanted their spouse to be in the room while using the VR. Although we did not collect data from the spouses, they appeared to experience joy watching their loved one engage in the VR with their adult child. In one of the cases, the spouse wanted to try the VR himself at the end of the study and his other son came to the senior living community to try it with him. The entire family benefited from the study, either directly or indirectly.

*Family members* ranged in age from 18 to 83 (*M =* 59.86, *SD* = 14.12), and included more males than females (nine females, 12 males). The majority (*n =* 17) were adult children, but the sample also included adult siblings (*n =* 3) and one godson. Six of the family members lived across the country and the rest lived in different parts of California, at least 45 min driving distance from the senior living community. On average, family members talked to their loved one once a week and visited them every 2 months in the senior living community. Only two of the family members were the primary caregivers for the resident. Most of the family members had siblings who were geographically closer to the resident who were the primary caregivers. On average, the residents reported being very close to the family member (*M* = 4.03, *SD* = 0.61 on a 5-point Likert scale) participating in this study and were satisfied with that relationship (*M* = 5.00, *SD* = 0.72 on a 6-point Likert scale) at baseline. Every participant was compensated $100.

### Recruitment and Informed Consent

The study was first approved by the Full Institutional Review Board. A letter was mailed to eligible families explaining the study, inviting them to participate, and allowing them to opt out. The staff then announced the study to residents. The researchers also attended townhalls and talked to the residents one-on-one. If the resident was interested, the researcher conducted the MMSE-2 and gathered contact information for the adult child who lived at a distance. If there was more than one adult child who lived at a distance, the researchers randomly chose one of the adult children to participate. Three of the participants did not have adult children but wanted a sibling or godson (who was considered a son) to participate instead. Potential participants were screened for hallucinations, paranoia, aggression, vertigo, or an abusive relationship with the family member. Formal consent was gathered from everyone at the start of the baseline survey. Residents with dementia were reconsented every time they used the VR and consent was obtained from legal representatives. If they had dementia, the researcher verbally went through the most important parts of the consent form one at a time and asked the resident to indicate that they understood each point before using the VR. During this process, the family member could also listen through the headset and offer assistance explaining the study if needed. Having the family member in the study with them provided a type of shared consent. For the residents with moderate dementia, three of them had spouses with them who also provided consent before every VR session. There were no issues with the consent process.

### Procedures

Resident–family member dyads completed a baseline telephone conversation (T1), followed by weekly VR sessions for 3 consecutive weeks (T2–T4) (see Table 1). Each time point was spaced 1 week apart. All of the sessions occurred before 5 p.m. to avoid sundowning. The families were asked to pick a time and day to do the study that worked best for them during a couple of set days during the week or on the weekend. Typically, the day and time were determined by the family members’ work schedule. The same day and time were scheduled every week for the 4 weeks. The residents experienced the VR in a private room at the senior living community, accompanied by the researcher who operated the VR using a control tablet. The participants used an Oculus Go, a standalone 3 degree-of-freedom VR headset with a single fast-switch LCD resolution of 2560×1440 and refresh rate of 60 Hz, powered by a Qualcomm Snapdragon 821 CPU mobile processor running an Android-based operating system. Family members participated from their own homes (see Supplementary Figure 2).

**Table 1.** Study Design and Timeline

Session details	Baseline (T1)	Week 1 (T2)	Week 2 (T3)	Week 3 (T4)
Title	Baseline phone call	Virtual adventures	Virtual travel life story	Virtual family photos and videos
Description	Resident and family member had a 15-min telephone conversation as they normally would.	Resident and family member chose five travel adventures among 25 possible preprogrammed adventures.	Resident and family member were taken back to favorite addresses or destinations from the past.	Resident and family member viewed their photos and video in a virtual family room, seated beside each other on a virtual couch. The resident chose avatars to represent themselves and their family member in the virtual world.
Data collection method	Survey	Survey/coding/interview	Survey/coding/ interview	Survey/coding/ interview
Mean minutes of session	17.67	23.93	33.65	33.49

*Note*: Participants in both mild cognitive impairment and Alzheimer’s disease/Alzheimer’s disease-related dementia groups engaged in an identical set of sessions.

All participants completed brief surveys, which occurred verbally if they had dementia, at the end of the baseline call and each VR session. Family members were emailed a Qualtrics link to the online surveys. They were also mailed the VR headset ahead of time, along with handouts about the study and reminders (e.g., plugging in the headset overnight). A researcher also called the family member and provided guidance on reminiscence therapy and technical assistance (e.g., setting up WiFi). The VR experience was personalized to each resident’s history and hobbies. To begin each VR session, the family member simply turned on their headset and put it on their eyes, which immediately allowed them to communicate with the resident through the built-in microphones in the headset.

In the *Baseline Session*, residents and their family member had a 15-min telephone conversation. They were instructed to talk to each other as they normally would during telephone conversations. In VR Session 1 (*Virtual Adventures*), the dyad chose five travel adventures (e.g., safari, hot air balloon, boat ride in Thailand) among 25 possible preprogrammed adventures. In Session 2 (*Virtual Life Story*), the researcher took residents and their family member back to favorite addresses or destinations from the past (e.g., childhood homes, family vacation sites, schools the resident attended). The family member input the addresses into the Rendever online portal. For Session 3 (*Virtual Photos and Videos*), the family member uploaded family photos and a video onto a password-protected, online portal. During this session, the resident and family member viewed their photos and video in a virtual family room, seated beside each other on a virtual couch. The resident also chose avatars to represent themselves and their family member in the virtual world. Although the residents with moderate dementia likely did not understand the purpose of the avatars, they enjoyed choosing their hair, eye, and skin color. This process took approximately 3 min.

The order of the sessions was designed so that the first two sessions did not require any extra effort on the part of the family member, and allowed them time to upload photos, videos, and addresses. The average length of the VR sessions was 30.36 min (range = 9–66 min; see Table 1 for means). We briefly interviewed the residents, in person, and family members, via the telephone, after each session to improve the VR. All of the sessions with the residents were videotaped and all participants completed every aspect of the study (there was no attrition).

Several improvements were made to the technology to improve the user experience. Even though the WiFi was tested beforehand with Rendever, one of the family members’ internet connections was weak, which disrupted the flow of the VR experience. This family member was mailed a hotspot for the second VR session, which solved the problem. After that session, we assessed the WiFi signal strength of each family member and mailed another one of the family members a hotspot, which prevented any further connection issues. We also had difficulties getting the preprogrammed video adventures to play immediately the first day of the VR sessions because the headsets were not shut down properly and did not receive a necessary update. From that point forward, we reminded everyone the day before their VR session to properly plug in the headsets. Finally, a couple of the videos that family members uploaded in the third VR session overloaded the system (after the family photos were already co-viewed and the session was coming to an end). This problem was solved by limiting the size of the videos that family members could upload to the family portal (Table 2).

**Table 2.** Mean User Experience Ratings for Residents

	VR session			
Scale items	Virtual adventures	Virtual life story	Virtual photos and videos	Overall
I am satisfied with the virtual experience	9.24 (1.14)	9.24 (1.41)	9.14 (1.71)	9.21 (1.42)
I am interested in the virtual experience	9.15 (1.39)	8.95 (1.86)	9.10 (2.10)	9.06 (1.78)
The virtual experience was easy to use	8.90 (2.41)	8.62 (2.50)	9.48 (1.57)	9.00 (2.19)
I felt secure when participating in the virtual experience	9.38 (1.96)	8.38 (3.22)	9.57 (1.17)	9.11 (2.30)
How much fun was the virtual reality system?	9.33 (1.07)	8.86 (1.91)	8.90 (2.21)	9.03 (1.78)
If it is available, how likely are you to consider using the virtual reality in the future?	8.52 (2.09)	8.05 (3.26)	8.71 (2.41)	8.43 (2.61)
How likely are you to recommend to a friend or family member that they try this virtual reality in the future?	8.58 (2.46)	8.67 (2.39)	8.71 (2.39)	8.62 (2.38)
The virtual experience was uncomfortable	2.14 (2.46)	1.38 (1.53)	1.14 (0.36)	1.56 (1.71)
The virtual experience irritated my eyes	1.43 (1.54)	1.48 (1.25)	1.29 (0.56)	1.40 (1.17)
The virtual experience gave me anxiety	1.14 (0.66)	1.19 (0.68)	1.19 (0.51)	1.17 (0.61)
The virtual experience made me feel tired	1.05 (0.22)	1.14 (0.36)	1.33 (1.11)	1.17 (0.69)
To what degree did you feel nauseous during the virtual experience?	1.29 (1.10)	1.24 (0.89)	1.10 (0.30)	1.21 (0.83)

*Notes*: VR = virtual reality. Values in parentheses are standard deviations. Response scales ranged from 1 to 10 with higher scores representing greater agreement or endorsement of the item. User experiences did not differ across the three VR sessions.

### Self-Report Measures and Coding of Videotapes

#### User satisfaction and perceptions of the VR

After each VR session, participants were asked about satisfaction, interest, ease of use, feelings of security using the VR, discomfort, eye irritation, anxiety, and fatigue with separate items along a 10-point scale (1 = *not at all*, 10 = *extremely*). These single items were adapted from Manera and colleagues’ (2016) study with older adults with dementia using VR. Two additional items were added: “The virtual experience irritated my eyes” and “The virtual experience was easy to use” along the same scale. Participants were also asked how much fun they had using the VR along a 1 (*not fun at all*) to 10 (*extremely fun*) scale (Maani et al., 2011). In addition, they were asked the extent they felt nauseous during the VR along a 1 (*no nausea at all*) to 10 (*vomit*) scale (Maani et al., 2011). In addition, we asked them if they would use the VR again, and if they would recommend it to a friend or family member along a 1 (*not at all likely*) to 10 (*extremely likely*) scale. Finally, we assessed three aspects of immersion in the VR sessions including *telepresenc*e (involvement and immersion in the environment; five items; α = .88), *social presence* (feeling of being with the other person; two items; *r* = .75), and *copresence* (degree to which each person was oriented toward the other person or involved with the other person in the VR; two items; *r* = .76), based upon scale items created by Nowak and Biocca (2003).

#### Observational coding of residents’ conversational and behavioral engagement

Residents’ engagement during the baseline telephone conversation and the VR sessions was coded from their videotaped verbal and nonverbal behavior. Four trained coders rated *Conversational engagement* (e.g., immediacy/affection, similarity/depth, receptivity/trust) with 19 items from the Relational Communication Scale (Burgoon & Hale, 1984). *Behavioral engagement* was rated with a modified version of Guerrero’s (2005) nonverbal coding items that measured *vocalics* (11 items; e.g., excited, animated voice), *facial expressions* around the mouth (five items; e.g., smiling, frowning, laughing), and *kinesics* (eight items; e.g., exaggeration of gestures, limb and trunk movement). All ratings ranged from 1 (*strongly disagree*) to 5 (*strongly agree*). Interrater reliabilities, established with 25% of the data, were excellent (.85 conversational engagement; .95 vocalics; .87 facial expressions; .82 kinesics) (see Supplementary Material: “Measures for Immersion” for all items and intercorrelations of the rated variables).

#### Automated coding of kinesic engagement

*OpenPose*, a free and open source body recognition software program, was used to code residents’ kinesic movements. The software can automatically detect human body, hand, facial, and foot keypoints (135 total keypoints) on single video images (Cao et al., 2017). Residents’ entire bodies were videotaped during the VR and initial telephone call, and then the distance of seven 2D coordinate points (i.e., nose, neck, shoulders, elbows, hands/wrists) between frames calculated as $\sqrt{{\left(x_{t+1}-x_{t}\right)}^{2}+{\left(y_{t+1}-y_{t}\right)}^{2}}$ was summed to obtain the information of each participant movement. For missing values of the coordinates (mainly due to occlusion), spline interpolation using the “na.spline” function of the *zoo* package in R was applied. The time series data of bodily movement was submitted to the wavelet transform to assess residents’ engagement (Grinsted et al., 2004). Because older adults’ movements tend to be slow, a wavelet spectrum analysis was performed to determine the rate of coding (the frequency band) that would best capture residents’ kinesic engagement. The most accurate frequency band (the region where the wavelet power was statistically significant against red noise backgrounds) was 0.5–1.5 Hz (a rhythm of once every 0.67–2 s). This frequency was used in all analyses (see Supplementary Figure 2 for illustrations). Automated coding of *kinesic engagement* was uncorrelated with human coding of *conversational engagement* (*r*_81_ = −.03, *p* = .819), *vocalics* (*r*_81_ = −.05, *p* = .665), *facial expressions* (*r*_81_ = −.04, *p* = .693), or *kinesics* (*r*_81_ = .14, *p* = .198).

## Results

### Statistical Analysis

Descriptive statistics were computed for all outcome variables at each time point. Separate analyses were conducted for residents and family members. To determine if user satisfaction differed across the three VR sessions, we computed repeated measures analysis of variance (ANOVA). To compare residents and family members, we used paired *t* tests. To determine if residents were more engaged during the VR sessions compared to the baseline telephone call, we conducted repeated measures ANOVAs on the *behavioral* measures of engagement. Finally, we conducted between-subjects ANOVAs to compare mean levels of each outcome for residents with MCI (*n* = 9) and mild or moderate dementia (*n* = 12). All tests for significance for the self-report and human coding were two-tailed, with α = .05. However, given the number of data points involved in the automated coding, Shaffer’s modified sequentially rejective Bonferroni procedure was used to correct for type 1 error.

### Residents’ and Family Members’ User Satisfaction With the VR Sessions

As shown in Table 3, residents were highly satisfied with their VR experiences. Mean (overall) ratings of satisfaction (*M* = 9.21) and interest (*M* = 9.06) were high (on a 10-point scale). The VR sessions were rated as fun (*M* = 9.03) and easy to use (*M* = 9.0), and participants reported feeling very secure (*M* = 9.11). Residents reported that they would be highly likely to use VR again if it was available (*M* = 8.43), and to recommend it to a friend or family member (*M* = 8.62) (all scales range from 1 to 10). No adverse reactions to the VR were reported and no one had to have their headset removed. Residents reported low levels of overall discomfort (*M* = 1.56), eye irritation (*M* = 1.40), and nausea (*M* = 1.21). They also reported very low levels of feeling anxious (*M* = 1.17) or tired (*M* = 1.17) during their VR sessions. To determine if residents’ experiences differed across the three VR sessions, we computed a series of repeated measures ANOVAs. Results revealed no significant differences across sessions.

**Table 3.** Mean User Experience Ratings for Family Members

	VR session			
Scale items	Virtual adventures	Virtual life story	Virtual photos and videos	Overall
I am satisfied with the virtual experience	8.29 (2.31)	8.81 (1.60)	8.57 (1.75)	8.56 (1.89)
I am interested in the virtual experience	9.33a (0.86)	8.57b (1.60)	8.81b (1.12)	8.90 (1.25)
The virtual experience was easy to use	8.57 (2.29)	8.75 (1.65)	7.50 (3.25)	8.28 (2.50)
I felt secure when participating in the virtual experience	9.70 (0.47)	9.35 (0.93)	8.86 (2.22)	9.30 (1.45)
How much fun was the virtual reality system?*	8.81a (1.17)	8.14b (1.82)	8.38 (1.60)	8.44 (1.55)
If it is available, how likely are you to consider using the virtual reality in the future?*	8.43 (2.06)	8.62 (1.50)	8.19 (2.04)	8.41 (1.86)
How likely are you to recommend to a friend or family member that they try this virtual reality in the future?*	8.71 (1.88)	8.81 (1.21)	8.52 (1.86)	8.68 (1.65)
The virtual experience was uncomfortable	2.19 (1.29)	1.62 (0.87)	2.33 (1.71)	2.05 (1.35)
The virtual experience irritated my eyes	1.62 (0.81)	2.08 (1.25)	1.71 (1.23)	1.81 (1.11)
The virtual experience gave me anxiety	1.38 (0.67)	1.43 (0.98)	1.33 (0.97)	1.38 (0.87)
The virtual experience made me feel fatigued	2.00 (2.19)	1.76 (1.41)	1.43 (0.98)	1.73 (1.60)
To what degree did you feel nauseous during the virtual experience?	1.95 (1.60)	1.67 (1.15)	1.48 (0.93)	1.70 (1.25)

*Notes*: VR = virtual reality. Values in parentheses are standard deviations. Unless otherwise noted, response scales ranged from 1 to 10 with higher scores representing greater agreement or endorsement of the item. Within rows, means with different letters differ significantly from each other (*p* < .05). The * mean that these were new items added by the authors.

Table 3 reports the comparable findings for adult family members. Overall, family members reported high levels of satisfaction. Mean (overall) ratings of satisfaction (*M* = 8.56) and interest (*M* = 8.90) were high. The virtual experiences were rated as fun (*M =* 8.44) and easy to use (*M* = 8.28), and participants felt very secure (*M* = 9.30). Family members reported that they would be highly likely to use VR again if it was available (*M* = 8.41), and to recommend it to a friend or family member (*M* = 8.68). No adverse reactions to the VR were reported. Family members reported low levels of overall discomfort (*M* = 2.05), eye irritation (*M* = 1.81), and nausea (*M =* 1.70). They also reported very low levels of feeling anxious (*M* = 1.38) or fatigued (*M =* 1.73) during the VR experience.

To determine if user experiences differed across the three VR sessions, we computed a series of repeated measures ANOVAs and pairwise contrasts. Results revealed no significant differences across the VR sessions with two exceptions. Family members rated the first VR session (virtual adventures) as more interesting than the second (virtual life story) and third (virtual family photos) sessions (*F*_(2,42)_ = 5.23, *p* = .009), and they rated the first session as more “fun” than the second session (*F*_(2,42)_ = 4.10, *p* = .024). Overall, family members experienced the VR sessions as highly enjoyable and comfortable, although the first session was slightly more enjoyable than the others.

Finally, we compared user satisfaction (averaged over the three VR sessions) for residents and family members with paired *t* tests. There were no significant differences within dyads, although there were several trends. Compared to their family members, residents experienced somewhat greater satisfaction (*t*_(20)_ = 1.71, *p* = .102; *M* = 9.21 vs 8.56) and ease of use (*t*_(20)_ = 1.77, *p* = .091; *M* = 9.00 vs 8.28) and slightly less fatigue (*t*_(20)_ = −1.69, *p* = .107; *M* = 1.17 vs 1.73) and nausea (*t*_(20)_ = −1.77, *p* = .092; *M* = 1.21 vs 1.70).

### Residents’ and Family Members’ Self-Reported Immersion During the VR Sessions

As shown in Table 4, residents and family members were highly immersed in their virtual environments and interactions with each other. Residents’ mean ratings of telepresence were above 4 on a 5-point scale for all VR sessions (overall *M* = 4.32). Social presence was also high (overall *M* = 3.95). Importantly, residents’ sense of copresence was uniformly high across VR sessions (overall *M* = 4.68). Repeated measures ANOVAs revealed no significant differences in residents’ self-reported immersion across the VR sessions.

**Table 4.** Mean Immersion Ratings for Residents and Family Members

	VR session			
Type of VR presence	Virtual adventures	Virtual life story	Virtual photos and videos	Overall
Residents				
Telepresence	4.31 (0.78)	4.23 (0.80)	4.42 (0.75)	4.32 (0.77)
Social presence	3.79 (1.06)	3.88 (1.19)	4.17 (1.03)	3.95 (1.09)
Copresence	4.64 (0.65)	4.62 (0.50)	4.79 (0.51)	4.68 (0.56)
Family members				
Telepresence	3.74 (0.78)	3.59 (0.90)	3.60 (1.04)	3.64 (0.90)
Social presence	3.14 (1.01)	3.45 (0.96)	3.45 (0.97)	3.35 (0.98)
Copresence	4.21 (0.58)	4.21 (0.70)	4.33 (0.60)	4.25 (0.62)

*Notes*: VR = virtual reality. Values in parentheses are standard deviations. Response scales ranged from 1 to 5 with higher scores representing greater endorsement of the item. User experiences did not differ significantly across VR sessions.

A similar pattern emerged for family members (see Table 4), although their overall means were slightly lower than for residents. Family members reported high levels of telepresence (overall *M* = 3.64), social presence (overall *M* = 3.35), and copresence (overall *M* = 4.25). Repeated measures ANOVAs revealed no significant differences in family members’ self-reported immersion across the three VR sessions.

Finally, we compared self-reported immersion for residents and family members with paired *t* tests. Compared to family members, residents experienced significantly greater telepresence (*t*_(20)_ = 3.23, *p* = .004), social presence (*t*_(20)_ = 2.69, *p* = .014), and copresence (*t*_(20)_ = 3.54, *p* = .002). Based upon our interview data, some of the family members wished the VR was more immersive and that the video images had better resolution. They also wished they could have seen their loved one using the VR inside the headset.

### Residents’ Behavioral Engagement During the Baseline Phone Call and VR Sessions

Residents’ engagement during all four sessions was assessed through human coding of conversational and physical engagement, and automated (computerized) coding of kinesics (see Table 5). A repeated measures ANOVA revealed significant differences in conversational engagement across sessions (*F*_(3,60)_ = 5.02, *p* = .004, partial η ^2^ = .20). The Virtual Life Story and Virtual Photos/Videos sessions (the sessions with reminiscence therapy) were significantly more conversationally engaging than the baseline telephone call and Virtual Adventures session. There were also significant differences in vocalics (*F*_(3,57)_ = 5.06, *p* = .004, partial η ^2^ = .21), facial expressions, (*F*_(3,57)_ = 3.00, *p* = .038, partial η ^2^ = .14), and kinesics (*F*_(3,57)_ = 17.25, *p* < .001, partial η ^2^ = .48). All three VR sessions were rated as more vocally and kinesically engaging than the baseline telephone call; and the Virtual Adventures and Virtual Family Photos/Videos sessions were rated as more facially engaging than the baseline telephone call. In addition, the Virtual Adventures and Virtual Life Story sessions were rated as more kinesically engaging than the Virtual Photos/Videos session. Finally, we found significant differences in automated coding of kinesics (*F*_(3,57)_ = 4.86, *p* = .004, partial η ^2^ = .20). The Life Story and Virtual Photos/Videos sessions were more engaging than the Virtual Adventures session and the baseline phone call.

**Table 5.** Mean Conversational and Behavioral Engagement for Residents

Type of coding	Baseline phone conversation	Virtual adventures	Virtual life story	Virtual photos and videos
Human coding of conversational engagement	4.74a (0.29)	4.66a (0.33)	4.84b (0.20)	4.86b (0.19)
Human coding of behavioral engagement—vocalics	4.61a (0.58)	4.82b (0.38)	4.80b (0.28)	4.83b (0.31)
Human coding of behavioral engagement—facial expressions	4.21a (0.79)	4.52b (0.62)	4.45 (0.45)	4.48b (0.53)
Human coding of behavioral engagement—kinesics	3.42a (0.77)	4.24b (0.69)	4.25b,c (0.50)	3.94d (0.55)
Automated coding of behavioral engagement—kinesics	0.028a (0.016)	0.039a (0.019)	0.045b (0.016)	0.045b (0.022)

*Notes*: Values in parentheses are standard deviations. *N* = 21 for conversational engagement, *N =* 20 for all other variables. Within rows, means with different letters differ significantly from each other (*p* < .05).

### User Experiences and Engagement for Residents With MCI and Dementia

To determine if residents with different levels of cognitive impairment differed in their responses to the VR, we conducted between-subjects ANOVAs comparing residents with MCI (*n* = 9) to those with mild-to-moderate dementia (*n* = 12) on all variables assessed during the VR sessions (see Table 6). There were no significant differences in user satisfaction, ease of use, or discomfort, indicating that residents with MCI and mild-to-moderate dementia equally enjoyed the VR and found it comfortable and easy to use. However, there were differences on all three self-report measures of immersion. On average, residents with dementia reported significantly greater telepresence (*F*_(1,19)_ = 4.73, *p* = .043, partial η ^2^ = .20), social presence (*F*_(1,19)_ = 15.92, *p* = .001, partial η ^2^ = .46), and copresence (*F*_(1,19)_ = 14.31, *p* = .001, partial η ^2^ = .43) than residents with MCI. There were no group differences in human coding of conversational, vocal, facial, or kinesic engagement. However, there was a difference in automated coding of kinesic engagement (*F*_(1,19)_ = 4.50, *p* = .047, partial η ^2^ = .19). On average, residents with MCI, compared to those with dementia, showed more kinesic engagement during the VR sessions.

**Table 6.** Mean Differences in All Outcome Variables for Residents With MCI and Dementia

Residents’ outcome variable	Level of cognitive impairment	
	MCI (*n =* 9)	Dementia (*n =* 12)
I am satisfied with the virtual experience	8.74 (1.61)	9.56 (0.56)
I am interested in the virtual experience	8.74 (1.71)	9.33 (0.88)
The virtual experience was easy to use	8.81 (1.54)	9.14 (1.75)
I felt secure when participating	9.52 (0.69)	8.81 (2.04)
How much fun was the virtual reality?	8.48 (2.07)	9.44 (0.84)
Likely to consider using in the future?	7.44 (3.10)	9.17 (1.50)
Likely to recommend?	7.96 (2.61)	9.11 (1.60)
Virtual experience was uncomfortable	2.04 (1.64)	1.19 (0.67)
Virtual experience irritated my eyes	1.63 (1.18)	1.22 (0.48)
Virtual experience gave me anxiety	1.30 (0.68)	1.08 (0.21)
Virtual experience made me feel tired	1.33 (0.67)	1.06 (0.13)
To what degree did you feel nauseous?	1.41 (0.72)	1.06 (0.13)
Telepresence	4.00a (0.76)	4.56b (0.38)
Social presence	3.22a (0.90)	4.49b (0.55)
Copresence	4.39a (0.42)	4.90b (0.18)
Human coding of conversational engagement	4.76 (0.26)	4.81 (0.26)
Human coding of behavioral engagement—vocalics	4.53 (0.26)	4.45 (0.58)
Human coding of behavioral engagement—facial expressions	4.81 (0.15)	4.81 (0.38)
Human coding of behavioral engagement—kinesics	4.21 (0.43)	4.09 (0.69)
Automated coding of behavioral engagement—kinesics	0.049a (0.018)	0.039b (0.019)

*Notes*: MCI = mild cognitive impairment. Values in parentheses are standard deviations. Means represent the average across the three virtual reality sessions (i.e., baseline excluded). Within rows, means with different letters differ significantly from each other (*p* < .05).

## Discussion

Almost no research has examined whether VR can enhance the social relationships of older adults, irrespective of their cognitive impairment (cf. Lin et al., 2018). The current study provides initial, strong evidence for the feasibility and technological merit of using VR with residents with varying levels of cognitive impairment and their family members who live at a distance. Residents and family members alike found the VR to be safe, extremely satisfying, and easy to use. In addition, the VR was acceptable and enjoyable for residents with MCI and mild-to-moderate dementia. Human and automated coding revealed that residents were more conversationally and behaviorally engaged with their family member in the VR sessions compared to the baseline telephone call and in the VR sessions that used reminiscence therapy. The results also illustrate the importance of using multiple methods to assess engagement. Residents with dementia reported greater immersion in the VR than residents with MCI. However, the automated coding indicated that residents with MCI were more kinesically engaged while using VR than residents with dementia. The results advance research on dementia (e.g., Sheehan et al., 2020; Sury et al., 2013) by providing an innovative tool that could potentially help reduce the emotional burden of ADRD for older adults and for those who love and care for them. The implications of the findings are detailed below.

### Using VR With Older Adults and Family Members From a Distance

The primary goal of this feasibility study was to test the remote capabilities and networking features of VR with older adults with cognitive impairments and their family members. Results demonstrate not only that the long-distance and networking features work effectively, but that both residents and family members loved engaging in the VR experiences with one another. The high levels of satisfaction and enjoyment, combined with the lack of adverse reactions and attrition, provide evidence of the acceptability of the VR.

Most research on VR only examines individual older adults’ satisfaction, cognitions, and emotions on isolated tasks (e.g., Appel et al., 2020; Manera et al., 2016; Repetto et al., 2016). What is unique about the current study is older adults and family members engaging in the VR experiences *together* from a distance. Our findings provide a foundation for future research on shared social experiences and relationship maintenance through VR. Maintaining important family connections, especially relationships with adult children, plays a crucial role in older adults’ mental health in senior living communities (Umberson et al., 2010). This is especially important during situations such as the COVID-19 pandemic when physical isolation restricts family visitations.

We also examined differences in enjoyment and acceptability across the VR sessions. Family members found the first VR session, which included more traditional VR travel adventures, the most fun and interesting. This finding could be due to the novelty of it being the first VR session or because the VR adventures involved more active movement inside the videos compared to the other sessions. The residents and family members also found the VR technology easy to use, even though the residents found it easier to use than the family members. This difference was likely due to the researcher managing the entire experience for the resident and the family member being responsible for some of the preparation for the VR sessions (e.g., configuring the WiFi on the headset; locating and uploading family photos and videos; identifying addresses). The residents reported enjoying all of the VR sessions equally. However, the human coding of conversational engagement revealed that residents were the most conversational with their family member during the VR sessions that took them back in time (i.e., Life Story and Family Photos/Videos) compared to the VR travel adventures or the baseline telephone call. The human coding (vocally, facially, and kinesically) and automated coding (kinesically) found similar behavioral engagement in those sessions compared to the baseline.

The aforementioned results validate previous research on the power of reminiscence therapy for engaging older adults (Lazar et al., 2014; Siverová & Bužgová, 2018). Numerous studies have discovered that reminiscence therapy can be used in VR to enhance cognition and memory (Chapoulie et al., 2014; Ferguson et al., 2020). However, this is the first study to use reminiscence therapy in VR with older adults and their social relationships. The current investigation is important because older adults in senior living communities often report high levels of depression, anxiety, and social isolation (Burgener et al., 2015), their adult children might not live near them anymore, and engagement with family members is a primary way these older adults can thrive (Moyle et al., 2011).

Residents and family members also reported high levels of immersion. Older adults, in particular, reported high levels of telepresence, social presence, and copresence. Because of its immersive nature, VR can allow older adults to feel as if they are actually inside the virtual world with their family member (see Biocca et al., 2003; Blascovich & Bailenson, 2011). In addition, when human coders rated the VR interaction as higher in conversational engagement, residents reported greater telepresence (*r*_61_ = .34, *p* = .007) and social presence (*r*_61_ = .25, *p* = .051). This could help promote and maintain secure attachments with family, which often become disrupted with the move into a senior living community (Sury et al., 2013). This might be especially important for older adults with dementia and their family members, who often feel as if dementia has “stolen” their memories, future life experiences, identity, and time together. The older adults also reported greater immersion in the VR than family members. It is important to remind family members that this VR platform is designed to be somewhat less immersive than other platforms so that it can be used safely and comfortably with older adults.

With the feasibility of the VR established, future research is necessary that examines the impact of this VR platform on the quality of life of the older adults and their family members. Older adults in senior living communities with dementia often rely heavily on their adult children for their sense of security and social connection (Moyle et al., 2011). Engaging in shared VR experiences together could improve the quality of life of the older adult and their adult child by experiencing their own and the other person’s joy using it. A larger study that is longer in duration (e.g., 3 months, 6 months) where the VR is used more frequently with family could shed light on whether the technology helps reduce caregiver guilt, decreases symptoms of depression and anxiety, and improves quality of life. Most research focuses on how VR helps the older adult, but it could be equally powerful for the family member as a result of the shared experience.

### VR and Older Adults’ Cognitive Impairment

Another goal of the current investigation was to determine the level of cognitive impairment for which the VR is most acceptable. The results revealed that the VR was safe, easy to use, fun, and enjoyable for all of the residents, regardless of their level of cognitive impairment. There were, however, slight differences in immersion and engagement depending upon whether the older adults had MCI or dementia. Using a multimethod approach (e.g., self-reports, human coding, automated coding) was important because it shed light on subtle, but important, differences in engagement based upon the level of cognitive impairment.

The automated coding revealed that residents with less cognitive impairment (or better MMSE-2 scores) were more behaviorally engaged in the VR than residents with more cognitive impairment. One plausible explanation for this finding is that as dementia becomes more severe, sometimes older adults’ bodily movements become more restricted (Tolea et al., 2016). These results have important implications for how researchers measure engagement, as well as practical implications for how people view older adults with dementia. For instance, adult children might think their parent who has dementia is not enjoying the VR (or another activity) if their parent is not very physically engaged in it. However, the older adults’ kinesics might not represent what they are feeling. In fact, there was not a significant correlation between physical engagement (coded from videos) and self-reported immersion. There could also be greater variability in older adults’ physical engagement using VR if they have dementia, compared to if they have MCI, and it is important not to equate physical engagement with emotional engagement.

The findings from the current study point to the importance of triangulating data to provide a more complete assessment of older adults’ outcomes (see also Allore et al., 2020). Collecting reliable and valid data from older adults with dementia can be incredibly challenging. It could be that the older adults with dementia in our study were able to answer survey questions because they had less severe symptoms of dementia (and still resided in independent living or assisted living rather than a nursing home or acute care hospital setting), and/or did not have as many other compounding health challenges as some older adults. We were able to collect self-report data from them by using decision trees (e.g., starting with agree or disagree and branching out), simplified scales and wording of items, visual cues, and by conducting the surveys verbally immediately after the VR. However, we also relied on survey data from the family member, observational coding (human and automated), and interviews with the older adult and family member. Together, the triangulation of various data collection techniques provided a holistic assessment of the older adults’ experiences with the VR.

The triangulation of data also revealed numerous practical guidelines for the use of the VR technology with older adults. Based upon the results of the current investigation, it is clear that the VR is safe and easy to use with older adults with dementia and that older adults with varying levels of cognitive impairment enjoy using it with their family members who live at a distance. Behavioral engagement while using the VR also varied by the level of cognitive impairment. For some older adults, their dementia might impair their behavioral engagement, but they are likely still engaged psychologically and emotionally. Family members also benefit from using the VR with their loved ones.

### Final Thoughts

The contributions of this study must be set within its limitations. Because this is a feasibility study, the sample size is rather small. A larger sample would have allowed for more comparisons, and potentially more power to find significant differences, between older adults with MCI and dementia. The dyads also only engaged in three VR sessions over the course of 3 consecutive weeks. Additional VR sessions and follow-up assessments are necessary to examine the long-term impact of the VR. Moreover, the researchers in this study operated the control tablet that dictated the VR experience. When used in senior living communities, activity directors or other trained staff would operate the VR equipment and software for their residents. Future research should determine the extent to which family caregivers can also be trained to safely use the VR with their loved ones with dementia at home. In addition, even though this technology can be used as an innovative tool to help older adults maintain important family relationships from a distance, the social nature of it could add an extra layer of logistics for senior living communities and families. However, given that adult children are typically the key decision makers in determining their parent’s care within a senior living community and they have a strong desire to improve the quality of that care (Sury et al., 2013), it would likely be worth the investment if the adult child and the parent are happier as a result of using the VR together.

Although technology is widely used to enable social connections, major barriers often emerge when it is adapted for the aging demographic, especially for those on the memory care spectrum. The VR technology tested here is unique in that it is specifically designed to take all major adoption barriers into account, while empowering caregivers and staff to create and deliver an authentic social experience. Older adults never have to actually interact with the physical technology, removing any barrier to adoption from them. With no technological barrier to entry, there is low risk of residents being unable to participate, which is essential when considering the potential impact of a social shared experience. The platform is also designed in a way that means that staff not only get to manage the devices and the experiences, but they are also equipped with staff-facing tools that promote the social experience; the technology helps to mediate the experience as well as the connection.

Finally, a 2D video might have been a better baseline than a telephone call to evaluate changes in engagement and satisfaction. These data were collected in the months prior to COVID-19, when the telephone was still the standard mode of communication with family in senior living communities. During COVID-19, it is likely that many older adults in senior living communities were taught how to use various video technologies (e.g., Zoom) to communicate with their family members from a distance. Future research should compare the current VR technology and its long-distance features with active control groups such as video chat. Even though video chat has similar features, the immersive nature of VR should allow for greater kinesic, psychological, and emotional engagement. Moreover, traveling and communicating in person are ideal. However, many older adults, especially if they have dementia, can no longer travel. VR technologies such as Rendever allow older adults with varying levels of cognitive impairment to continue to live life and to travel back in time with family members, who might be geographically separated from them.

The results from this study show that the long-distance feature of the VR is effective and that it can be used safely with older adults with mild-to-moderate dementia. The next step is to determine whether this VR technology can influence the quality of life of older adults with varying levels of cognitive impairment and their family members who live at a distance. Combining networking and livestreaming features in a single VR platform can allow older adults in senior living communities to still travel, relive their past, and engage fully with life *with* their family members, despite geographical separation and physical and cognitive challenges.

## Supplementary Material

igab014_suppl_Supplementary_Materials

## References

[CIT0001] Allore, H. G., Goldfeld, K. S., Gutman, R., Li, F., Monin, J. K., Taljaard, M., & Travison, T. G. (2020). Statistical considerations for embedded pragmatic clinical trials in people living with dementia. Journal of the American Geriatrics Society, 68(suppl. 2), S68–S73. doi:10.1111/jgs.16616 32589276 PMC7396162

[CIT0002] Angelini, L., Caon, M., Couture, N., Khaled, O. A., & Mugellini, E. (2015). The multisensory interactive window: Immersive experiences for the elderly. UBICOMP/ISWC, 15, 7–11. doi:10.1145/2800835.2806209

[CIT0003] Appel, L., Appel, E., Bogler, O., Wiseman, M., Cohen, L., Ein, N., Abrams, H. B., & Campos, J. L. (2020). Older adults with cognitive and/or physical impairments can benefit from immersion virtual reality experiences: A feasibility study. Frontiers in Medicine, 6, 329–339. doi:10.3389/fmed.2019.00329 32010701 PMC6974513

[CIT0004] Appel, L., Kisonas, E., Appel, E., Klein, J., Bartlett, D., Rosenberg, J., & Smith, C. (2020). Introducing virtual reality therapy for inpatients with dementia admitted to an acute care hospital: Learnings from a pilot to pave the way to a randomized controlled trial. Pilot and Feasibility Studies, 6, 166. doi:10.1186/s40814-020-00708-9 33292729 PMC7602317

[CIT0041] Benoit, M. (2015). Is it possible to use highly realistic virtual reality in the elderly? A feasibility-based study with image based rendering. Neuropsychiatric Disease and Treatment, 11, 557–563. doi:10.2147/NDT.S73179 25834437 PMC4357614

[CIT0005] Biocca, F., Harms, C., & Burgoon, J. (2003). Toward a more robust theory and measure of social presence: Review and suggested criteria. Presence, 12, 456–480. doi:10.1162/105474603322761270

[CIT0006] Blascovich, J., & Bailenson, J. N. (2011). Infinite reality—Avatars, eternal life, new worlds, and the dawn of the virtual revolution. William Morrow.

[CIT0007] Burgener, S. C., Bukwalter, K., Perkhounkova, Y., & Liu, M. F. (2015). The effects of perceived stigma on quality of life outcomes in persons with early stage dementia: Longitudinal findings: Part 2. Dementia, 14, 609–632. doi:10.1177/1471301213504202 24339117

[CIT0008] Burgoon, J., & Hale, J. (1984). The fundamental topoi of relational communication. Communication Monographs, 51, 193–214. doi:10.1080/03637758409390195

[CIT0009] Cagle, J. G., & Munn, J. C. (2012). Long-distance caregiving: A systematic review of the literature. Journal of Gerontological Social Work, 55, 682–707. doi:10.1080/01634372.2012.703763 23078605 PMC5653258

[CIT0010] Cao, Z., Simon, T., Wei, S. E., & Sheikh, Y. (2017). Realtime multi-person 2D pose estimation using part affinity fields. In Proceedings of the IEEE Conference on Computer Vision and Pattern Recognition (pp. 7291–7299).

[CIT0011] Centers for Disease Control and Prevention. (2020). https://www.cdc.gov/chronicdisease/about/multiple-chronic.htm

[CIT0012] Chapoulie, E., Guerchouche, R., Petit, P. D., Chaurasia, G., Robert, P., & Drettakis, G. (2014). Reminiscence therapy using image-based rendering in VR. IEEE Virtual Reality, March 29–April 2. doi:10.1371/journal.pone.0151487

[CIT0013] Cummings, J. J., & Bailenson, J. N. (2016). How immersive is enough? A meta-analysis of the effect of immersive technology on user presence. Media Psychology, 19, 272–309. doi:10.1080/15213269.2015.1015740

[CIT0014] Derrick, D. C., Jenkins, J. L., & Nunamaker, J. F. (2011). Design principles for Special Purpose, Embodied, Conversational Intelligence with Environmental Sensors (SPECIES) agents. AIS Transactions on Human-Computer Interaction, 3, 62–81. https://aisel.aisnet.org/thci/vol3/iss2/2/

[CIT0015] Eriksen, S., Helvik, A., Juvet, J. K., Skovdahl, K., Foresund, H., & Gov, E. K. (2016). The experience of relations with persons with dementia: A systematic meta-synthesis. Dementia and Geriatric Cognition Disorders, 42, 342–368. doi:10.1159/000452404 27866199

[CIT0016] Ferguson, C., Shade, M. Y., Blaskewicz Boron, J., Lyden, E., & Manley, N. A. (2020). Virtual reality for therapeutic recreation in dementia hospice care: A feasibility study. The American Journal of Hospice & Palliative Care, 37, 809–815. doi:10.1177/1049909120901525 31975609

[CIT0017] Grinsted, A., Moore, J. C., & Jevrejeva, S. (2004). Application of the cross wavelet transform and wavelet coherence to geophysical time series. Nonlinear Processes in Geophysics, 11, 561–566. doi:10.5194/npg-11-561-2004

[CIT0018] Guerrero, L. K. (2005). Observer ratings of nonverbal involvement and immediacy. In V. Manusov (Ed.), The sourcebook of nonverbal measures: Going beyond words (pp. 221–235). Lawrence Erlbaum.

[CIT0040] Harms, C., & A. Biocca, F. (2004). Internal consistency and reliability of the networked minds social presence measure. In M. Alcaniz & B. Rey (Eds.), Seventh Annual International Workshop: Presence 2004. Valencia: Universidad Politecnica de Valencia.

[CIT0019] Jensen, M. L., Lee, Y., Piercy, C. W., Dunbar, N. E., Elizondo, J., Bessarabova, E., Twyman, N. W., Burgoon, J. K., Valacich, J. S., Adame, B., Miller, C., & Wilson, S. (2016). Exploring failure and engagement in a complex digital training game: A multi-method examination. AIS Transactions on Human-Computer Interaction, 8, 1–20. doi:10.17705/1thci.08102

[CIT0020] Koenig, T. L., Lee, J. H., & MacMillon, K. R. (2014). Older adult and family member perspectives of the decision-making process involved in moving to assisted living. Qualitative Social Work, 13, 335–350. doi:10.1177/1473325013475468

[CIT0021] Lazar, A., Thompson, H., & Demiris, G. (2014). A systematic review of the use of technology for reminiscence therapy. Health Education & Behavior, 41(suppl. 1), 51S–61S. doi:10.1177/1090198114537067 25274711 PMC4844844

[CIT0022] Lin, C. X., Lee, C., Lally, D., & Coughlin, J. F. (2018). Impact of virtual reality (VR) experience on older adults’ well-being. In J. Zhou & G. Salvendy (Eds.), Human aspects of IT for the aged population. Applications in health, assistance, and entertainment. ITAP 2018. Lecture Notes in Computer Science, vol. 10927. Springer.

[CIT0042] Maani, C. V., Hoffman, H. G., Morrow, M., Maiers, A., Gaylord, K., McGhee, L. L., DeSocio, P. A.. (2011). Virtual reality pain control during burn wound debridement of combat-related burn injuries using robot-like arm mounted VR goggles. The Journal of Trauma, 71, S125–S130. doi:10.1097/TA.0b013e31822192e2 21795888 PMC4460976

[CIT0026] Manera, V., Chapoulie, E., Bourgeois, J., Guerchouche, R., David, R., Ondrej, J., Drettakis, G., & Robert, P. (2016). A feasibility study with image-based rendered virtual reality in patients with mild cognitive impairment and dementia. PLoS One, 11, e0151487. doi:10.1371/journal.pone.0151487 26990298 PMC4798753

[CIT0027] McEwen, D., Taillon-Hobson, A., Bilodeau, M., Sveistrup, H., & Finestone, H. (2014). Two-week virtual reality training for dementia: Single case feasibility study. Journal of Rehabilitation Research and Development, 51, 1069–1076. doi:10.1682/JRRD.2013.10.0231 25437527

[CIT0028] Moyle, W., Jones, C., Dwan, T., & Petrovich, T. (2018). Effectiveness of a virtual reality forest on people with dementia: A mixed methods pilot study. The Gerontologist, 58, 478–487. doi:10.1093/geront/gnw270 28329867

[CIT0029] Moyle, W., Venturto, L., Griffiths, S., Grimbeek, P., McAllister, M., Oxlade, D., & Murfield, J. (2011). Factors influencing quality of life for people with dementia: A qualitative perspective. Aging & Mental Health, 15, 970–977. doi:10.1080/13607863.2011.583620 22022878

[CIT0024] National Institutes of Health. (2017). What is Alzheimer’s disease? https://www.nia.nih.gov/health/what-alzheimers-disease

[CIT0025] Nowak, K., & Biocca, F. (2003). The effect of the agency and anthropomorphism on users’ sense of telepresence, copresence, and social presence in virtual environments. Presence, Telepresence, and Virtual Environments, 12, 481–494. doi:10.1162/105474603322761289

[CIT0030] Optale, G., Urgesi, C., Busato, V., Marin, S., Piron, L., Priftis, K., Gamberini, L., Capodieci, S., & Bordin, A. (2010). Controlling memory impairment in elderly adults using virtual reality memory training: A randomized controlled pilot study. Neurorehabilitation and Neural Repair, 24, 348–357. doi:10.1177/1545968309353328 19934445

[CIT0031] Repetto, C., Serino, S., Macedonia, M., Riva, G. (2016). Virtual reality as an embodied tool to enhance episodic memory in the elderly. Opinion, 7, 1–4. doi:10.3389/fpsyg.2016.01839 PMC5113123 27909424

[CIT0032] Sayma, M., Tuijt, R., Cooper, C., & Walters, K. (2020). Are we there yet? Immersive virtual reality to improve cognitive function in dementia and mild cognitive impairment. The Gerontologist, 60, e502–e512. doi:10.1093/geront/gnz132 31600389

[CIT0033] Sheehan, O. C., Haley, W. E., Howard, V. J., Huang, J., Rhodes, J. D., & Roth, D. L. (2020). Stress, burden, and well-being in dementia and nondementia caregivers: Insights from the caregiving transitions study. The Gerontologist. Advance online publication. doi:10.1093/geront/gnaa108 PMC8276607 32816014

[CIT0034] Siverová, J., & Bužgová, R. (2018). The effect of reminiscence therapy on quality of life, attitudes to ageing, and depressive symptoms in institutionalized elderly adults with cognitive impairment: A quasi-experimental study. International Journal of Mental Health Nursing, 27, 1430–1439. doi:10.1111/inm.12442 29427397

[CIT0035] Sury, L., Burns, K., & Brodaty, H. (2013). Moving in: Adjustment of people living with dementia going into a nursing home and their families. International Psychogeriatrics, 25, 867–876. doi:10.1017/S1041610213000057 23425369

[CIT0036] Thompsell, A., & Lovestone, S. (2002). Out of sight out of mind? Support and information given to distant and near relatives of those with dementia. International Journal of Geriatric Psychiatry, 17, 804–807. doi:10.1002/gps.692 12221652

[CIT0037] Tolea, M., Morris, J., & Galvin, J. (2016). Trajectory of mobility decline by dementia type. Alzheimer’s Disease Associated Disorders, 30, 60–66. doi:10.1097/WAD.0000000000000091 PMC4592781 25886717

[CIT0038] Umberson, D., Crosnoe, R., & Reczek, C. (2010). Social relationships and health behavior across life course. Annual Review of Sociology, 36, 139–157. doi:10.1146/annurev-soc-070308-120011 PMC3171805 21921974

[CIT0039] Vertesi, A., Lever, J. A., Molloy, D. W., Sanderson, B., Tuttle, I., Pokoradi, L., & Principi, E. (2001). Standardized Mini-Mental State Examination: Use and interpretation. Canadian Family Physician, 47, 2018–2023. https://www.ncbi.nlm.nih.gov/pmc/articles/PMC2018449/11723596 PMC2018449

