# Supplementary material for: Testing the Feasibility of Virtual Reality With Older Adults With Cognitive Impairments and Their Family Members Who Live at a Distance
Source: Innov Aging. 2021 Jul 1;5(2):igab014. doi: 10.1093/geroni/igab014 (PMC8494141; doi:10.1093/geroni/igab014)
Supplement: igab014_suppl_Supplementary_Materials [file igab014_suppl_supplementary_materials.docx]

**Online Supplementary Material**

**Supplementary Table 1. Characteristics of the sample**

| [MISSING] | Residents  (*N* = 21) | Adult Family Members  (*N* = 21) |
| --- | --- | --- |
| Female, *n* (%) | 18 (86%) | 9 (46%) |
| Age (years), range, *M* (*SD*) | 54–95, 83.1 (9.76) | 18–83, 59.86 (14.12) |
| Ethnicity, *n* (%)  White  Native American  Declined to state | 19 (90%)  1 (5%)  1 (5%) | 20 (95%)  –  1 (5%) |
| Level of education, *n* (%)  High School  AA Degree  Some College  BA Degree  MA Degree  PhD or other  Not specified | 2 (10%)  1 (5%)  3 (14%)  11 (52%)  2 (10%)  2 (10%)  – | 1 (5%)  2 (10%)  3 (14%)  10 (47%)  3 (14%)  1 (5%)  1 (5%) |
| MMSE2, range, *M* (*SD*) | 13–26, 22.19 (3.79) | – |
| Cognitive impairment, *n* (%)  MCI  Dementia | 9 (43%)  12 (57%) | –  – |
| Family member’s relationship  to the resident, *n* (%)  Adult child  Sibling  Godson | –  –  – | 18 (86%)  3 (14%)  1 (5%) |

*Notes*. MMSE2 is the Mini-Mental State Examination-2; M = Mean; SD = Standard Deviation

**Supplementary Figure 1. How Rendever works with older adult-family dyads**


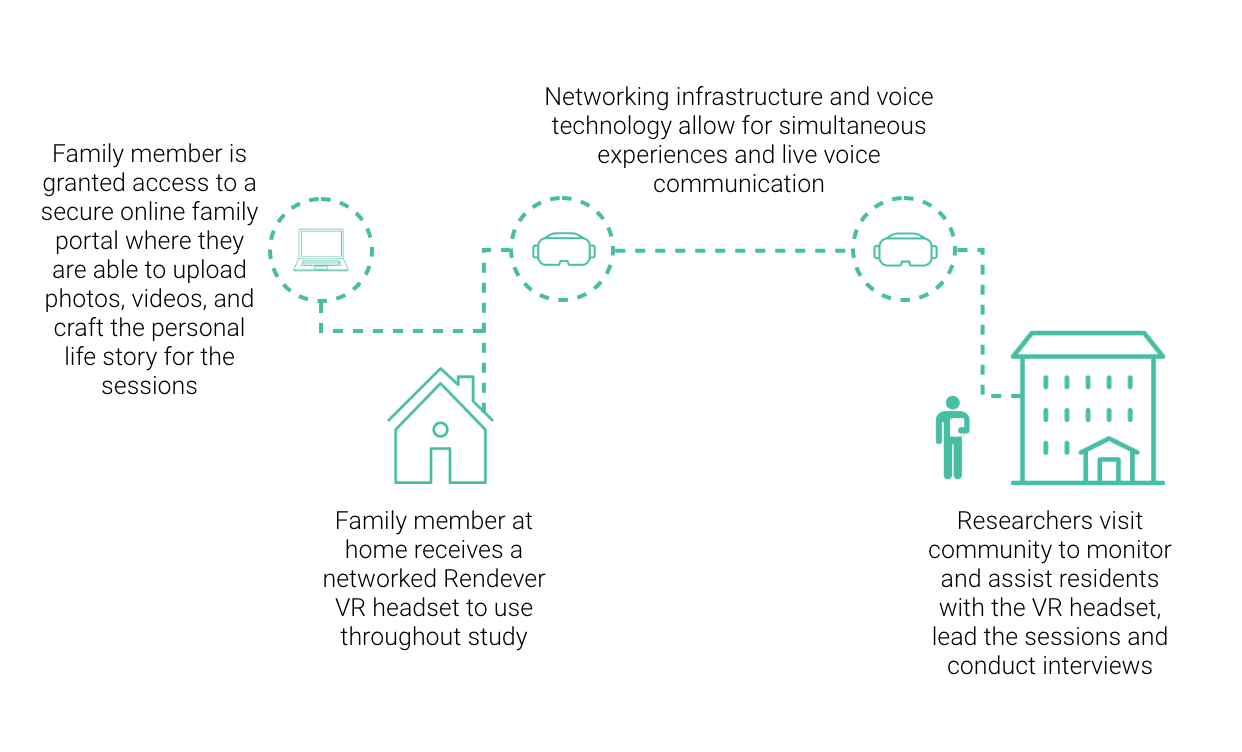


**Supplementary Figure 2. Example of automated coding of a resident using OpenPose and the wavelet transform of their movement time series**


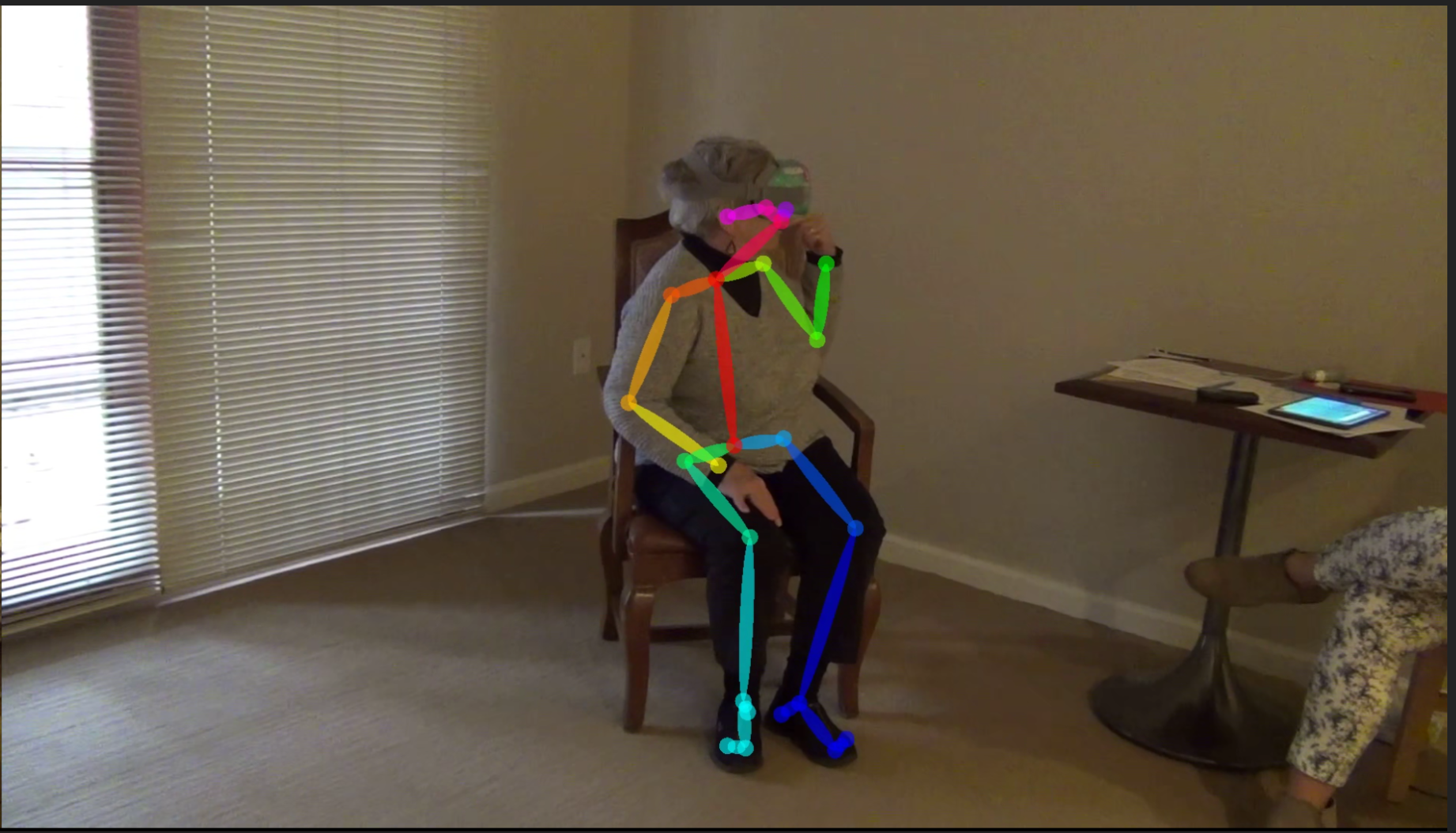

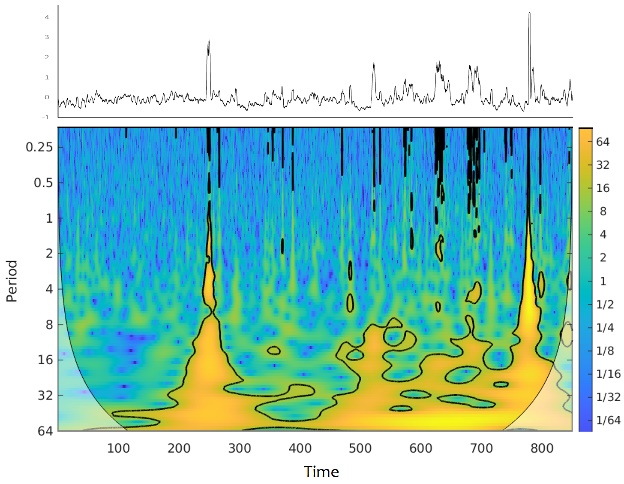


*Note*. The area where the wavelet power was significant against red noise backgrounds is surrounded by black lines. A period is an inverted value of the frequency band.

**Supplementary Document: Measures for Immersion**

**Immersion Measures**

*Telepresence:*

How involving was the experience?

How intense was the experience?

To what extent did you feel like you were inside the environment you saw/heard?

To what extent did you feel immersed in the environment you saw/heard?

To what extent did you feel surrounded by the environment you saw/heard?

*Social-presence*:

To what degree was this like being face-to-face with your child/parent?

To what degree did you feel like you were in the same room with your child/parent?

*Co-presence*:

My child/parent was intensely involved in our conversation.

My child/parent was interested in talking with me.

**Measures for Observational Coding**

**Conversational Engagement Items**

1. He/she was involved in the conversation.
2. He/she found the conversation positively stimulating.
3. He/she seemed to desire further communication between them.
4. He/she was interested in talking to the other person.
5. He/she communicated coldness rather than warmth.**
6. He/she was willing to listen to the other person.
7. He/she was calm and poised with the other person.
8. He/she tried to move the conversation to a deeper level.
9. He/she was comfortable interacting with the other person.
10. He/she acted bored by their conversation.**
11. He/she was relaxed talking with the other person.
12. He/she seemed nervous.**
13. He/she had a difficult time articulating his/her thoughts.**
14. He/she used long pauses between turn-taking.**
15. He/she was affectionate toward the other person.
16. His/her communication with the other person seemed comfortable.
17. He/she communicated a sense of happiness with the other person.
18. He/she expressed feelings of joy toward the other person.
19. He/she reminisced positive moments from the past with the other person.

Note. **Item is reverse-coded. Coders were asked to rate the extent to which the resident they were rating engaged in each behavior with their family member, using the following scale: (1) strongly disagree, (2) disagree, (3) neutral, (4) agree, and (5) strongly agree. These items were adapted from Burgoon and Hales’ (1987) Relational Communication Scale. Items 15-19 were original items added to this scale. All of the items were averaged to form one, overall measure of conversational engagement.

**Behavioral Engagement Items**

**Facial Expression**

Stem: Based on your observation of the resident’s face, the resident:

1. Always smiled
2. Was facially pleasant
3. Conveyed positive affect
4. Laughed a lot
5. Conveyed a sense of wonder or “awe”

**Vocalics**

Stem: The resident’s voice:

1. Contained vocal variety
2. Sounded relaxed
3. Sounded warm
4. Sounded calm
5. Was animated
6. Sounded pleasant
7. Was friendly
8. Was full of life
9. Was very excited
10. Sounded happy
11. Sounded content

**Kinesics**

Stem: The resident showed:

1. Very little nervous movement
2. Infrequent fearful behaviors
3. A lot of gesturing
4. A lot of kinesic expression
5. A lot of trunk/limb movement
6. Enthusiastic engagement in VR
7. A lot of initiation in VR

*Note*. The coders rated the extent to which the resident engaged in each behavior on the above items for facial expressions, vocalics and kinesics, using the following scale: (1) strongly disagree, (2) disagree, (3) neutral, (4) agree, and (5) strongly agree.
